# Association between dipeptidyl peptidase-4 inhibitors use and leptin in type 2 diabetes mellitus

**DOI:** 10.1186/s13098-021-00703-x

**Published:** 2021-08-26

**Authors:** Xin Wei, Yu Bai, Zhuo Wang, Xiaohong Zheng, Zening Jin, Xin Liu

**Affiliations:** 1 Department of Cardiology and Macrovascular Disease, Beijing Tiantan Hospital, Capital Medical University, Beijing, China; 2 Department of Otolaryngology, Beijing Haidian Hospital, Beijing, China; 3 Department of Gastroenterology, Beijing Tiantan Hospital, Capital Medical University, Beijing, China; 4 Department of Neuro-Oncology, Cancer Center, Beijing Tiantan Hospital, Capital Medical University, Beijing, China; 5 Department of Pharmacy, Beijing Tiantan Hospital, Capital Medical University, Beijing, China

**Keywords:** Dipeptidyl peptidase-4 inhibitors, Leptin, Type 2 diabetes mellitus, Randomized controlled trials

## Abstract

**Background:**

Dipeptidyl peptidase-4 inhibitors (DPP-4i) provide a unique antihyperglycemic effect by regulating incretin peptides in type 2 diabetes mellitus (T2DM) patients who are inadequately controlled with insulin therapy. The aim of this study was to investigate the impact of DPP-4i on leptin concentrations in subjects with T2DM.

**Methods:**

Randomized controlled trials (RCTs) with comparators were systematically searched through PubMed, Embase, and the Cochrane Library. Quantitative analysis was performed with a fixed or random-effects model according to heterogeneity. Publication bias was evaluated by using the standard methods for sensitivity analysis.

**Results:**

Ten trials with 698 patients with T2DM were included. Pooled analysis demonstrated that DPP-4i did not significantly change leptin concentrations (1.31 ng/mL, 95 % CI − 0.48 to 3.10). DPP-4i exerted effects on modulating leptin levels compared to active comparators (0.21 ng/mL, 95 % CI − 1.37 to 1.78). Meta-analysis was powerful and stable after sensitivity analysis.

**Conclusions:**

DPP-4i did not modulate leptin concentrations in T2DM and exerted no stronger effects than traditional antidiabetic agents.

**Supplementary Information:**

The online version contains supplementary material available at 10.1186/s13098-021-00703-x.

## Introduction

Type 2 diabetes mellitus (T2DM) is a common metabolic disease characterized by hyperglycemia and is often accompanied by obesity. Reports indicate that obesity is a promoter of T2DM and childhood-obesity increases the risk of T2DM in adulthood [1]. An obesity forecast study based on a nonlinear regression model suggested that 51 % of the population in the world will be obese by 2030 [2]. In the obese state, excessive visceral fat accumulation could cause adipose tissue dysfunctionality that contributes to the onset of obesity-related comorbidities [3]. The functions of adipose tissue include not only storing energy but also synthesizing and secreting adipocytokines. These adipokines play distinct roles in physiological and pathophysiological conditions. Among them, leptin is an adipokine mainly secreted by adipose tissue and serves as an afferent signal for maintaining homeostasis of adipose tissue mass [4]. Data have suggested that dysregulated leptin is usually associated with metabolic diseases, including obesity and T2DM [5]. Apart from obesity, hyperleptinemia is linked with insulin resistance and hypertension. In addition, leptin independently reduces blood glucose levels, particularly in hyperglycemic models of insulin deficiency [6]. Currently, a number of studies have shown that different antidiabetic agents modulate serum leptin concentrations in physiological and pathophysiological conditions [7–9].

Novel glucose-lowering drugs including sodium-glucose cotransporter 2 (SGLT2) inhibitors, glucagon-like peptide 1 (GLP-1) receptor agonists, and Dipeptidyl peptidase-4 inhibitors (DPP-4i) have become available. These agents provide protective effects by reducing blood glucose levels and improving insulin resistance [10, 11]. Effective glucose control by these new agents significantly improves long-term microvascular and macrovascular complications in patients with T2DM [10, 12]. Among them, dipeptidyl peptidase 4 inhibitors exert the effect on lowering blood glucose by inhibiting the inactivation of GLP-1 [13]. The efficacy and safety of DPP-4 inhibitors have been proved by several randomized controlled trials, demonstrating improved glucose control with a low risk of hypoglycemia [14]. It remains unknown whether DPP-4 inhibitors could modulate leptin and to what extent compared to other antidiabetic agents. Therefore, the current study aimed to help demonstrate the impact of DPP-4i on leptin levels in T2DM.

## Methods

### Search strategy

We searched PubMed, Embase, and the Cochrane Library for randomized controlled trials (RCTs) published in English from inception until 30 March 2021. The key terms used were “sitagliptin” OR “vildagliptin” OR “teneligliptin” OR “saxagliptin” OR “linagliptin” OR “alogliptin”. Two authors independently performed the literature search.

### Study selection

All RCTs lasting at least 4 weeks and reporting data on leptin outcome were included. A study was identified if it was a randomized controlled study comparing DPP-4i with placebo or other antidiabetic agents, if it reported leptin levels with DPP-4i treatment, and if it was conducted in patients with T2DM. A study was excluded if it was conducted in healthy participants, it was non-human designed, if it was a narrative review, or only an abstract paper. Reference lists of eligible studies as well as systematic reviews and meta-analyses of DPP-4i were hand-searched for additional relevant studies. Corresponding authors were contacted if relevant information was missing. Inclusion and exclusion criteria were evaluated objectively by two reviewers.

### Data extraction

Two authors independently extracted data by using standardized predefined forms : first author name, year of article publication, country origin, sample size, gender distribution, body mass index, mean age, diabetes duration, DPP-4 inhibitor(s), comparator(s), therapy duration, baseline glycated hemoglobin A1c(HbA1c), and serum leptin concentrations. The primary outcome measure was a change in leptin concentrations. When studies reported leptin data for different treatment durations, the longest was used.

### Quality evaluation

Study quality was assessed using the Cochrane risk of bias tool. The parameters included random sequence generation, allocation concealment, blinding of participants and personnel, blinding of outcome assessment, incomplete outcome data, selective outcome reporting, and other potential sources of bias. According to the Cochrane risk tool, “yes” indicated a low risk of bias, while “no” indicated a high risk of bias. “Unclear” indicated an unknown or unclear risk of bias.

### Statistical analysis

We undertook a meta-analysis using STATA version 14.1 (Stata Corp, College Station, TX, USA). Continuous leptin outcome was pooled with a fixed-effects or random-effects model according to study heterogeneity. The results were reported with 95 % confidence intervals (CIs), and P values < 0.05 were considered statistically significant. Heterogeneity was assessed using chi-squared tests and quantified with the *I*^*2*^ index. Sensitivity analysis was conducted with the leave-one-out method to assess the influence of each study on the overall effect size. Publication bias was examined by Begg’s test and Egger’s test if there were at least five studies in the meta-analysis. Subgroup analysis was performed according to treatment duration, age, BMI, leptin, and HbA1c at baseline.

## Results

### Study inclusion process and characteristics of included studies

**Fig. 1:**
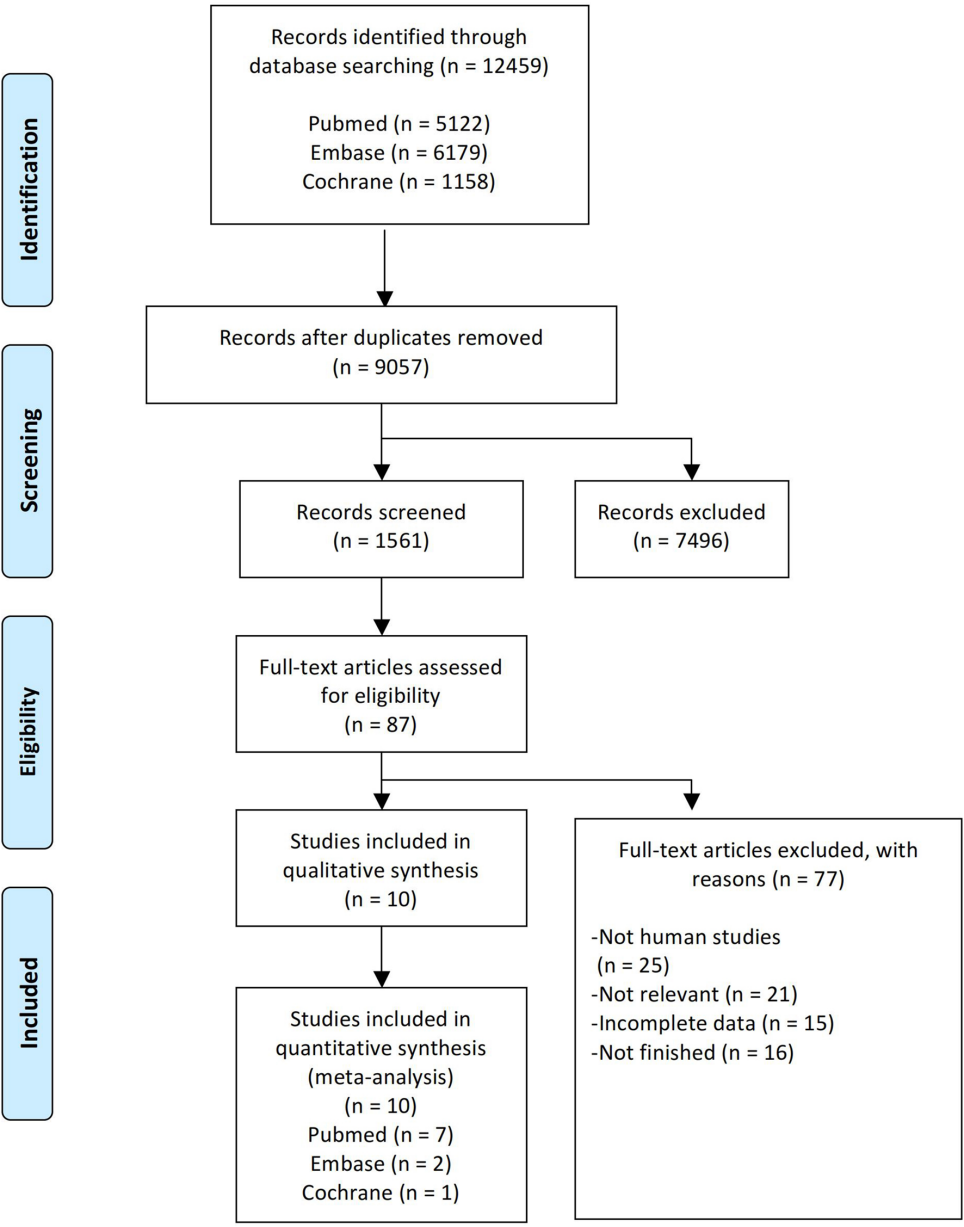
Flow diagram of the study selection process

From 12,459 identified records, we excluded non-human and observational studies, leaving 10 for full-text assessment. After systematic selection (Fig. 1), 10 RCTs fulfilled the inclusion criteria (Table 1). RCTs published between 2015 and 2021 included 698 participants. Of these, 348 were treated with a DPP-4 inhibitor (175 with sitagliptin, 72 with vildagliptin, 21 with saxagliptin, 42 with alogliptin, and 38 with linagliptin), monotherapy or in addition to metformin or other antidiabetic agents, and 350 were treated with placebo or control therapy. The follow-up time ranged from 1 to 13 months. In the largest study, 241 subjects were included, while the smallest one recruited 20 subjects. Most patients received sitagliptin, while the remaining studies compared vildagliptin, saxagliptin, alogliptin, and linagliptin with placebo or traditional antidiabetic agents, respectively.

**Table. 1:** Demographic characteristics of the studies included

Study, year	Location	Treatment arm (n)	HbA1c(%)	Male (n)	Age (years)	BMI (kg/m^2^)	Diabetes duration (years)	Treatment duration (months)	Leptin (ng/mL)
Takeshita [37]	Japan	Sitagliptin:28 Mitiglinide:29	6.7 ± 0.6 6.9 ± 0.8	18 19	61.0 ± 13.8 65.8 ± 9.7	24.5 ± 3.8 24.2 ± 4.6	86.4 ± 90 145.2 ± 122.4	4	8.7 ± 6.5 10.5 ± 13.4
Takeshita [38]	Japan	Vildagliptin:53 Liraglutide:49	8.1 ± 1.2 8.0 ± 0.9	36 35	64.5 ± 12.7 64.9 ± 1.9	24.5 ± 4.6 25.4 ± 4.8	NS	3	8.1 ± 6.9 6.9 ± 5.7
Kato [39]	Japan	Sitagliptin:10 Glimepiride:10	7.2 ± 0.2 7.3 ± 0.2	6 5	62 ± 4.7 55 ± 6.7	25.6 ± 2.6 26.6 ± 2.5	NS	6	12.6 ± 2.3 10.3 ± 3.0
Matsushima [40]	Japan	Sitagliptin: 120 Voglibose:121	7.9 ± 1.0 7.8 ± 0.8	72 71	63.2 ± 13.8 63.2 ± 11.6	25.0 ± 4.5 25.1 ± 4.5	NS	3	8.3 ± 6.9 9.0 ± 9.3
Dore [41]	American	Saxagliptin:21 Placebo:21	7.0 ± 0.8 6.6 ± 0.5	10 14	58.3 ± 5.7 56.4 ± 8.5	32.3 ± 4.2 31.5 ± 4.8	120	3	19.4 ± 3.7 14.1 ± 2.1
Takihata [42]	Japan	Sitagliptin:17 Luseogliflozin:17	10.0 ± 1.41 0.4 ± 1.0	14 15	52.8 ± 15.5 52.1 ± 15.3	26.8 ± 5.1 26.4 ± 4.8	NS	3	9.1 ± 6.7 7.2 ± 4.7
Takeshita [43]	Japan	Alogliptin:42 Metfomin:42	7.5 ± 1.07 .4 ± 1.2	29 29	63.8 ± 10.5 63.1 ± 13.1	25.4 ± 6.1 24.4 ± 4.0	122.4 ± 124.8 169.2 ± 156	3	11.2 ± 12.8 8.4 ± 10.7
Schiapaccassa [44]	Brazil	Vildagliptin:19 Metformin:19	8.0 ± 1.8 7.9 ± 2.0	0 0	39.1 ± 5.3 39.8 ± 7.7	36.0 ± 4.0 38.5 ± 6.1	NS	1	21.9 ± 19.4 25.4 ± 13.3
Awal [45]	American	Linagliptin:14 Placebo:17	7.1 ± 0.7 7.4 ± 1.0	11 7	61.0 ± 5.0 63.0 ± 6.0	31.2 ± 4.4 30.6 ± 2.9	≤ 180	3	21.7 ± 22.8 22.5 ± 12.6
Komorizono [46]	Japan	Linagliptin:24 Metformin:25	7.0 ± 0.5 7.2 ± 0.8	10 9	49.4 ± 10.8 55.6 ± 10.2	29.7 ± 4.9 27.9 ± 4.1	NS	13	17.7 ± 9.3 18.5 ± 8.2

### Quality evaluation

**Fig. 2:**
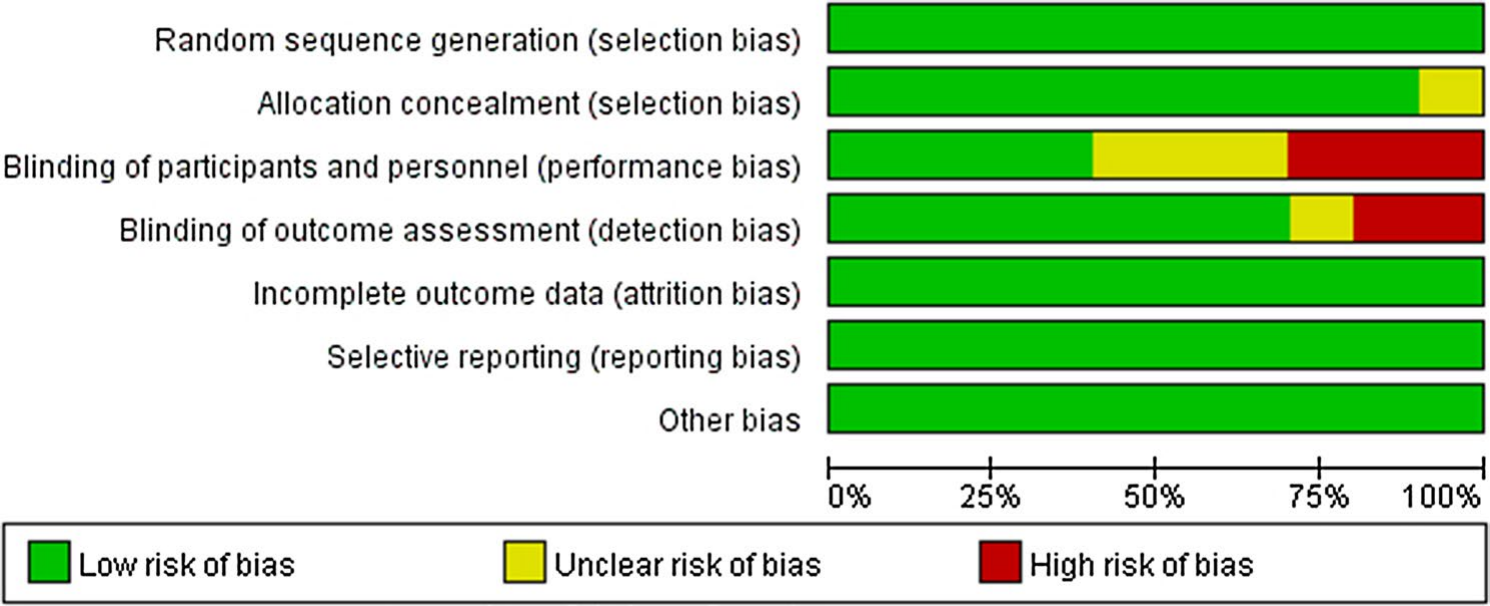
Risk of bias graph

The results of quality evaluation e displayed in Fig. 2. Overall, the risk of bias for the items was judged to be low. All the studies were randomly designed. There was an unclear risk of bias in some items, including allocation concealment, blinding of the outcome and participants. Two studies had detection bias based on blinding of outcome assessment, and three studies had performance bias due to the lack of implementation of blind methods.

### Meta-analysis of the effect of DPP-4i treatment

**Fig. 3:**
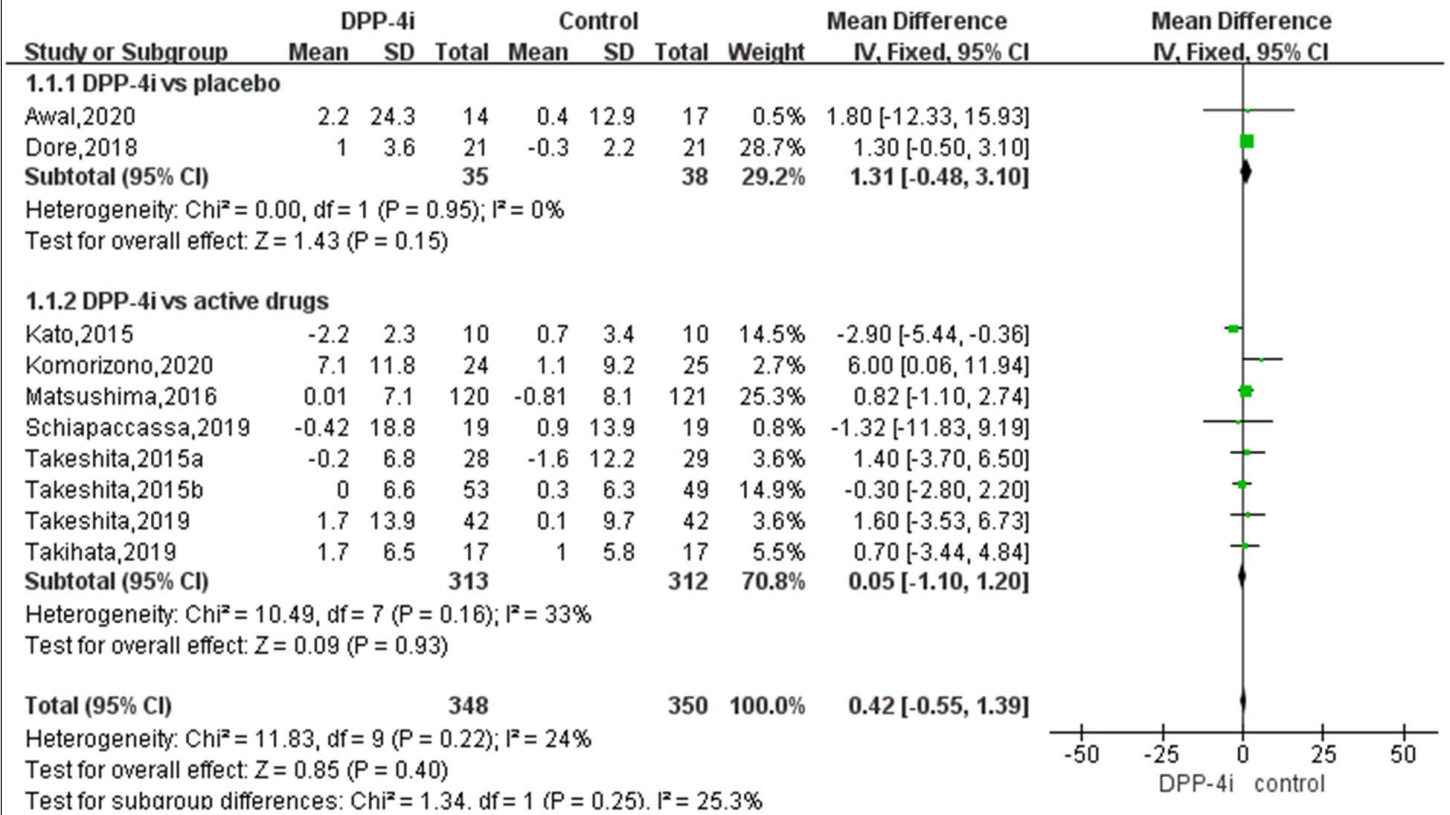
Meta-analyses of Dipeptidyl peptidase-4 inhibitors (DPP-4i) versus other anti-diabetic treatments or placebo

**Fig. 4:**
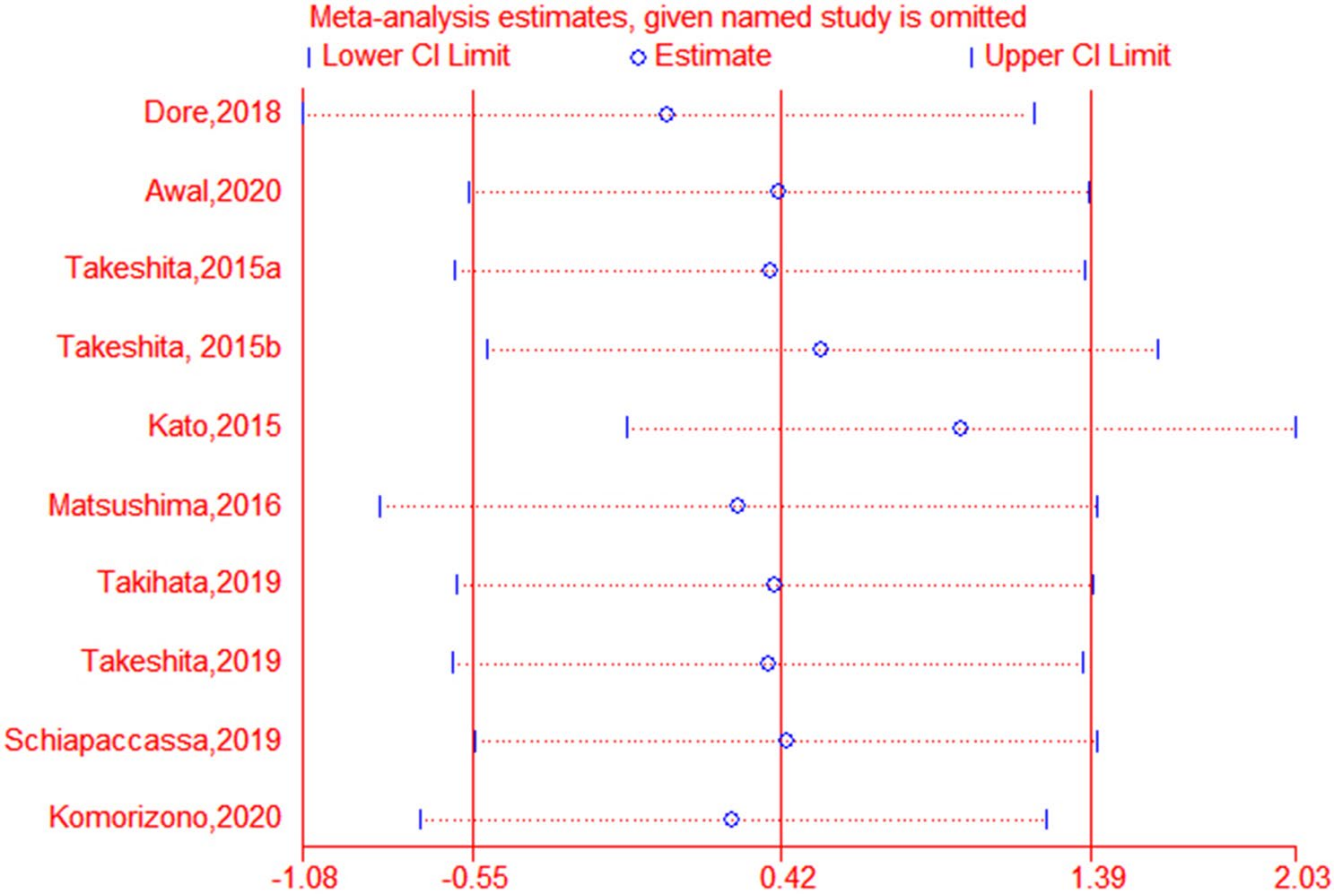
The graph of sensitivity analysis (The vertical line on the left indicates the total lower level of CI, the vertical line in the middle indicates the total pooled effect size, and the vertical line on the right indicates the total higher level of CI. The circle indicates the pooled effect size after deleting the study. The dotted line indicates the 95 % CI range after the deletion of the study.)

**Fig. 5:**
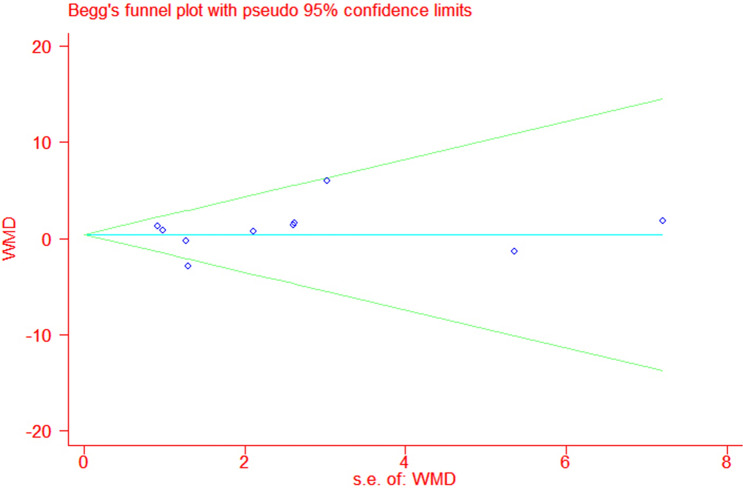
The funnel plot for the role of dipeptidyl peptidase-4 inhibitors in type 2 diabetes mellitus

Leptin data were available from all RCTs. Based on the pooled analysis, the effect of DPP-4i on leptin concentrations was 1.31 ng/mL (95 % CI, − 0.48 to 3.10, *P* = 0.95, *I*^*2*^ = 0 %) compared to placebo, and 0.21ng/mL (95 % CI, − 1.36 to 1.78, *P* = 0.16, *I*^*2*^ = 33 %) compared to traditional antidiabetic agents (Fig. 3). The pooled estimate of the modulating effect of DPP-4i on leptin was credible in the leave-one-out sensitivity analysis (WMD 0.42ng/mL, 95 % CI − 0.54, 1.39, N = 10 studies, heterogeneity *P* = 0.39; Fig. 4). This confirmed that the effect across the studies was an overall effect of all the identified studies. In the subgroup analysis, the baseline HbA1c, leptin, BMI, length of follow-up, andage parameters did not influence the impact of DPP-4i on leptin (see Additional file 1: Figs. S1–S5). No publication bias was suggested by Begg’s test (*P* = 0.93) or Egger’s test (*P* = 0.95) across the 10 studies (Fig. 5).

## Discussion

The present study aimed to evaluate the effect of DPP-4 inhibitors on serum levels of leptin in T2DMs. Leptin is associated with metabolism, insulin sensitivity, and diabetes. Interestingly, this meta-analysis demonstrated that DPP-4 inhibitors exerted no significant effect on changing circulating leptin levels in T2DM patients compared to placebo or active drugs.

Leptin is a protein containing 167 amino acids that is mainly secreted by white adipose tissue into the blood and can be transported across the blood-brain barrier [15]. Leptin plays a key role in regulating the physiologic switch between the fed and starved states. Since its discovery in 1994, leptin has provided deep insights into the regulation of central nervous system energy balance circuits [16]. This adipokine regulates metabolic homeostasis by inhibiting food intake and increasing energy expenditure. Surprisingly,leptin significantly reduced blood glucose in mouse models of insulin-deficient diabetes, suggesting that leptin modulated glucose homeostasis independently of insulin [17, 18]. Although its exact mechanism of lowering glucose levels remains unknown, data have shown that leptin decreases appetite, suppresses insulin secretion, and increases insulin sensitivity. Overall, the identification of leptin has provided a framework for studying the pathogenesis of obesity in the diabetic population. Decreases in sensitivity to leptin might contribute to the development of T2DM [19].

Leptin therapy has been found to effectively reverse hyperglycemia and prevent mortality in mouse models of diabetes [20]. The pathogenesis of diabetes is different from that of obesity, and reports have shown that it might be related to leptin hyposecretion or leptin resistance. The former type of diabetes is characterized by low endogenous plasma leptin levelsand these patients respond to leptin therapy, while the latter describes most obese subjects, who are leptin resistant but might respond to leptin therapy in combination with other drugs such as leptin sensitizers [21]. In the state of T2DM, leptin resistance was observed and leptin action was decreased in the brain parenchyma and vessels, despite its elevated concentrations in the plasma [18]. In T2DM, elevated levels of leptin are often linked with increased cardiovascular risks, as well as with the presence of macro- and microvascular complications. Diabetic subjects might benefit from correction of leptin resistance as well as insulin resistance [20]. In patients with severe coronary artery disease, abdominal obesity is commonly related to increased leptin concentrations and decreased adiponectin concentrations. Leptin/adiponectin imbalance might mediate the increased risks of developing T2DM and cardiovascular diseases(CVDs) associated with abdominal obesity [22]. Our team previously found that DPP-4 inhibitors increased serum adiponectin levels in T2DMs [23]. In the current study, we demonstrated that DPP-4 inhibitors did not significantly change serum leptin concentrations, suggesting that these drugs provided a neutral effect without aggravating leptin resistance in the diabetic state.

Although the cardiovascular safety of DPP-4 inhibitors has been proven in T2DM, the net effect of these drugs on leptin concentrations in obesity-related disease remains unclear [24]. In Kitamura’s study, leptin sensitivity was enhanced after anagliptin treatment in high-fat diet fed mice [25]. In the study evaluating the inhibitory effect of vildagliptin on fibrosis markers in white adipose tissue of high-fat diet-induced obese mice, vildagliptin prevented the increase of fibrosis markers and reduced leptin levels [26]. The effect of sitagliptin on reducing BMI and the occurrence of hypoglycemia in obese patients with insulin treatment-induced diabetes mellitus might be correlated with decreased leptin levels and increased adiponectin levels [27]. Add-on therapy with anagliptin in Japanese T2DM patients treated with metformin for 52 weeks also reduced leptin concentrations [28]. Our study included most studies with relatively shorter treatment durations lasting from 1 to 6 months, with only 1 study lasting for 13 months. Further studies with a longer duration and a larger number of participants will be needed to illuminate the effect of DPP-4 inhibitors on leptin concentrations and leptin sensitivity.

Besides, T2DM is associated with metabolic dysregulation and chronic inflammation. Data emerging from research on leptin in diabetes suggest that it is an inflammatory mediator that sustains multifactorial diseases [29]. Leptin induces tumor necrosis factor-α (TNF-α)-dependent inflammation in acquired generalized lipodystrophy disease [30]. Statins [31] and antidiabetic agents [32] including sitagliptin, metformin, pioglitazone, liraglutide, and empagliflozin exhibit certain effects on inflammation. Sitagliptin effectively improved diet-induced metabolic syndrome and fatty liver via regulation of adipose tissue inflammation and hepatic adiponectin/ leptin levels [33]. Another study proved that the novel DPP-4 inhibitor teneligliptin prevents high-fat diet-induced obesity accompanied by increased energy expenditure in mice [34]. DPP-4 inhibitor anagliptin exerts anti-inflammatory effects on macrophages, adipocytes, and mouse liver by suppressing NF-kB activation [35]. We also found that the inflammatory marker C-reactive protein was effectively reduced after DPP-4 inhibition [36]. Further data should be reviewed regarding the role of leptin in inflammation, and the role of inflammation in the development of leptin resistance and obesity.

Although there have been some reports investigating the effect of antidiabetic agents including DPP-4 inhibitors, no confirming answers on whether DPP4 inhibitors modulate leptin have been drawn. We could not obtain further information for other comparisons for modulating leptin levels in T2DMs. In the absence of comparative evidence between DPP-4i and other anti-diabetic medications, this meta-analysis added detailed illustration on adipokine of leptin levels. In this pooled analysis, comparisons of DPP-4i therapy and other treatment for type 2 diabetes (with 10 included trials) were performed, providing evidence that DPP-4i treatment was not significantly associated with changing leptin levels in participants from different regions in comparison with placebo. This effect of DPP-4i on leptin levels was not changed by potential variablesincluding treatment duration, age, and baseline HbA1c.

This study is the first meta-analysis demonstrating the effect of DDP-4i on serum leptin concentrations in T2DMs. It suggested that DPP-4i did not exert an effect on leptin resistance in T2DM patients with obesity-associated cardiovascular diseases. It also provides insights into the therapeutic implications of obesity-related atherosclerotic disease in humans and the potentially protective effects on leptin sensitivity. Secondly, the pooled results suggest that leptin potentially serves as an effective cardiovascular biomarker in T2DM. Thirdly, subgroup analysis was performed to explore the effect of therapy duration, diabetes duration, dosage, and age.

This study also has some limitations needed to be disclosed. Firstly, only studies published in English were searched, which could inevitably generate publication bias and unstable estimates of treatment effects. What’s more, the pooled analysis should be interpreted with consideration for the moderate heterogeneity across identified studies, although measures were alleviate it by performing the sensitivity analysis and subgroup analysis. Thirdly, the follow-up periods were relatively short, and evaluating the long-term effects of DPP-4i treatment is necessary.

## Conclusions

DPP-4i exerted no off-target effects on modulating leptin concentrations in patients with T2DM. Data on the long-term effects are needed to perform in patients with T2DM with risks of obesity and cardiovascular disease.

## Supplementary Information

**Additional file 1**:** Figure S1**. Subgroup analysis based on treatment duration.** Figure S2**. Subgroup analysis based on BMI.** Figure S3**. Subgroup analysis based on leptin.** Figure S4**. Subgroup analysis based on age. ** Figure S5**. Subgroup analysis based on HbA1c.

## Data Availability

All data generated or analyzed during this study are included in this published article [and its supplementary information files].
