# Supplementary material for: Association between dipeptidyl peptidase-4 inhibitors use and leptin in type 2 diabetes mellitus
Source: Diabetol Metab Syndr. 2021 Aug 26;13:88. doi: 10.1186/s13098-021-00703-x (PMC8390252; doi:10.1186/s13098-021-00703-x)
Supplement: Supplementary file 1 — Additional file 1: Figure S1. Subgroup analysis based on treatment duration. Figure S2. Subgroup analysis based on BMI. Figure S3. Subgroup analysis based on leptin. Figure S4. Subgroup analysis based on age. Figure S5. Subgroup analysis based on HbA1c. [file 13098_2021_703_MOESM1_ESM.docx]

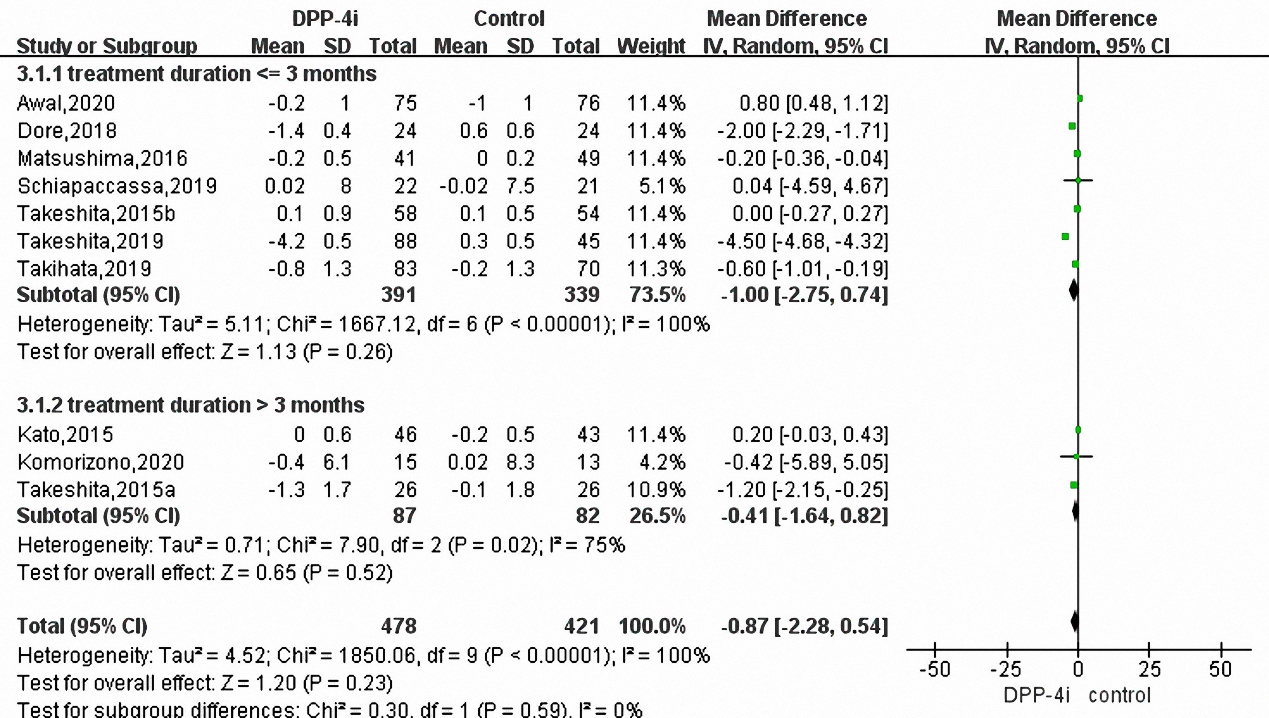


Supplementary figure 1 Subgroup analysis based on treatment duration.


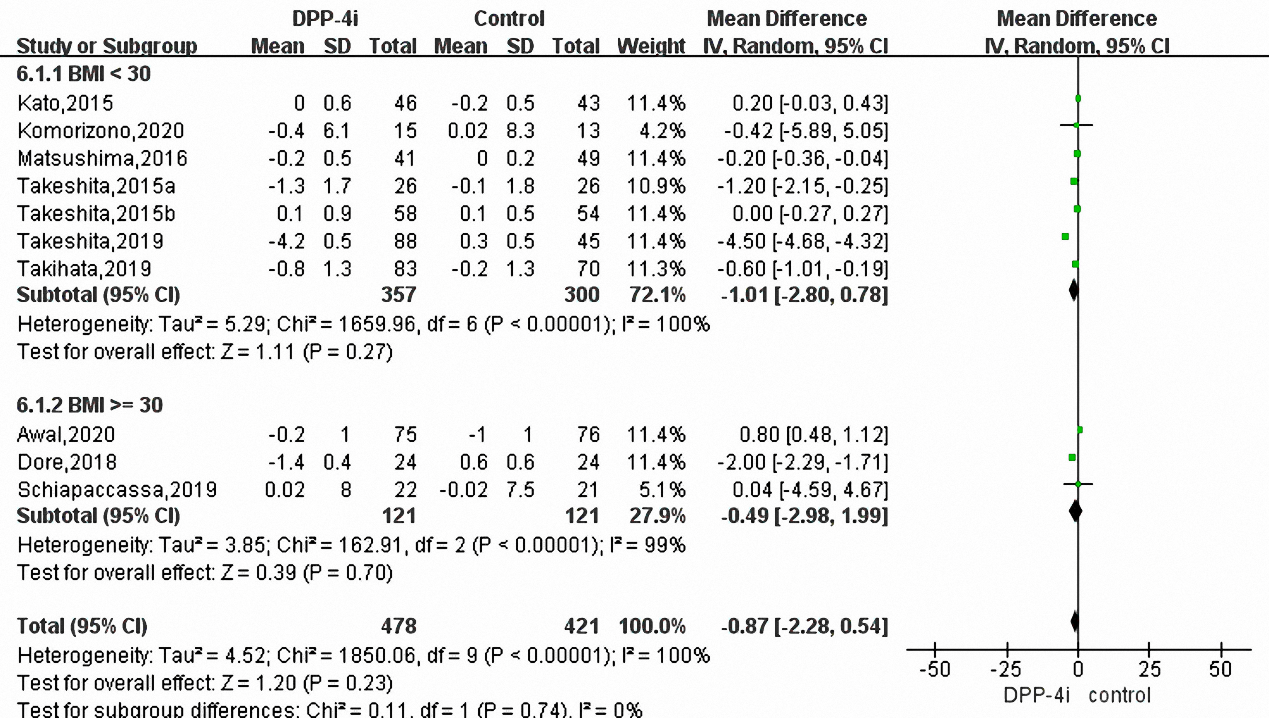


Supplementary figure 2 Subgroup analysis based on BMI.


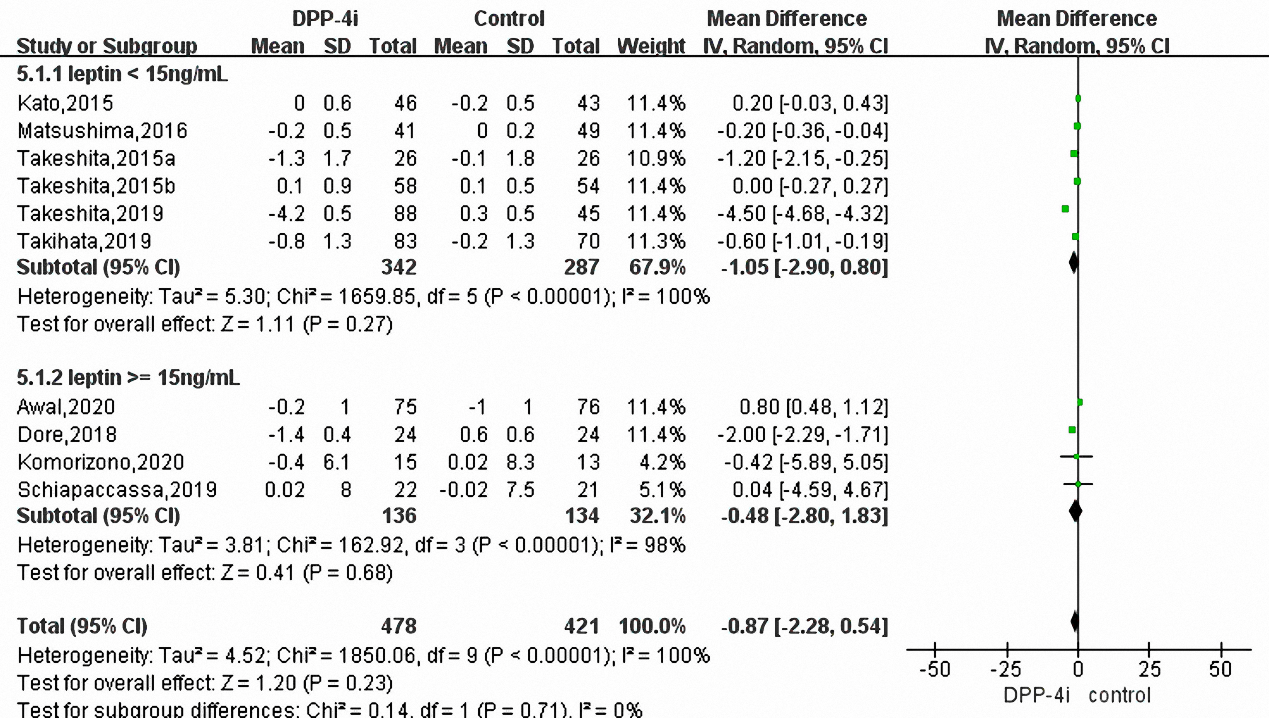


Supplementary figure 3 Subgroup analysis based on leptin.


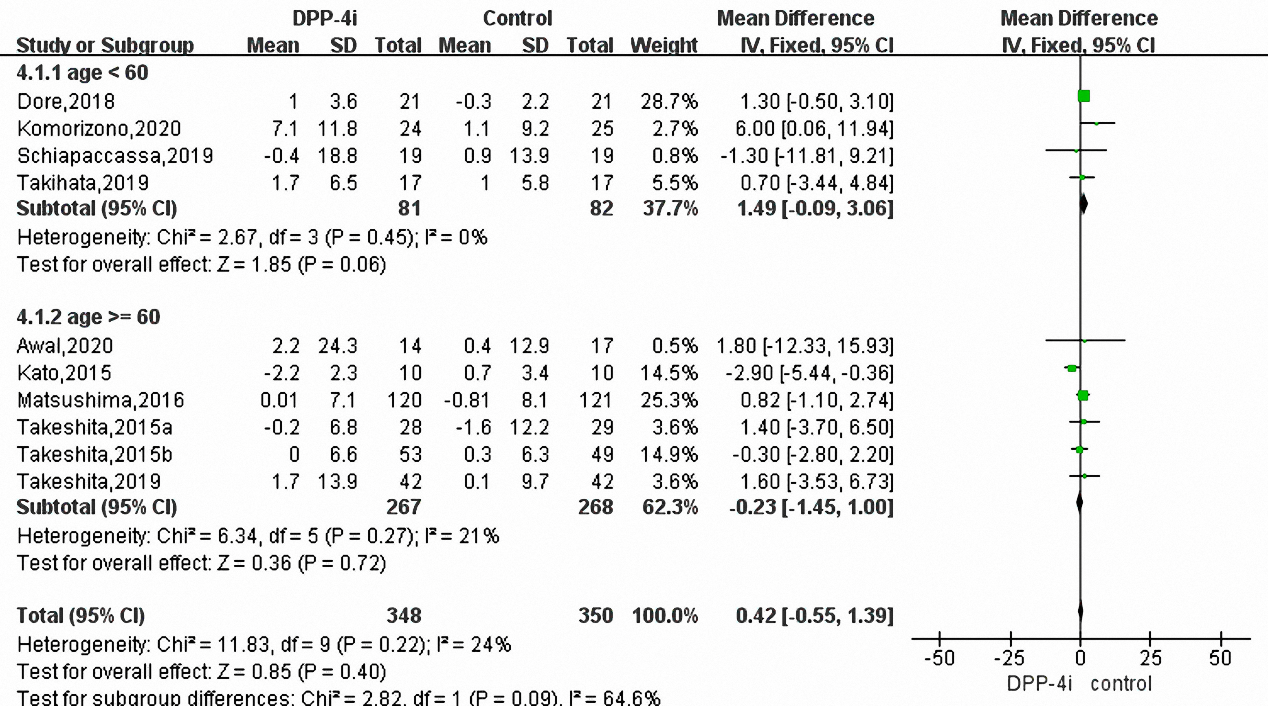


Supplementary figure 4 Subgroup analysis based on age.


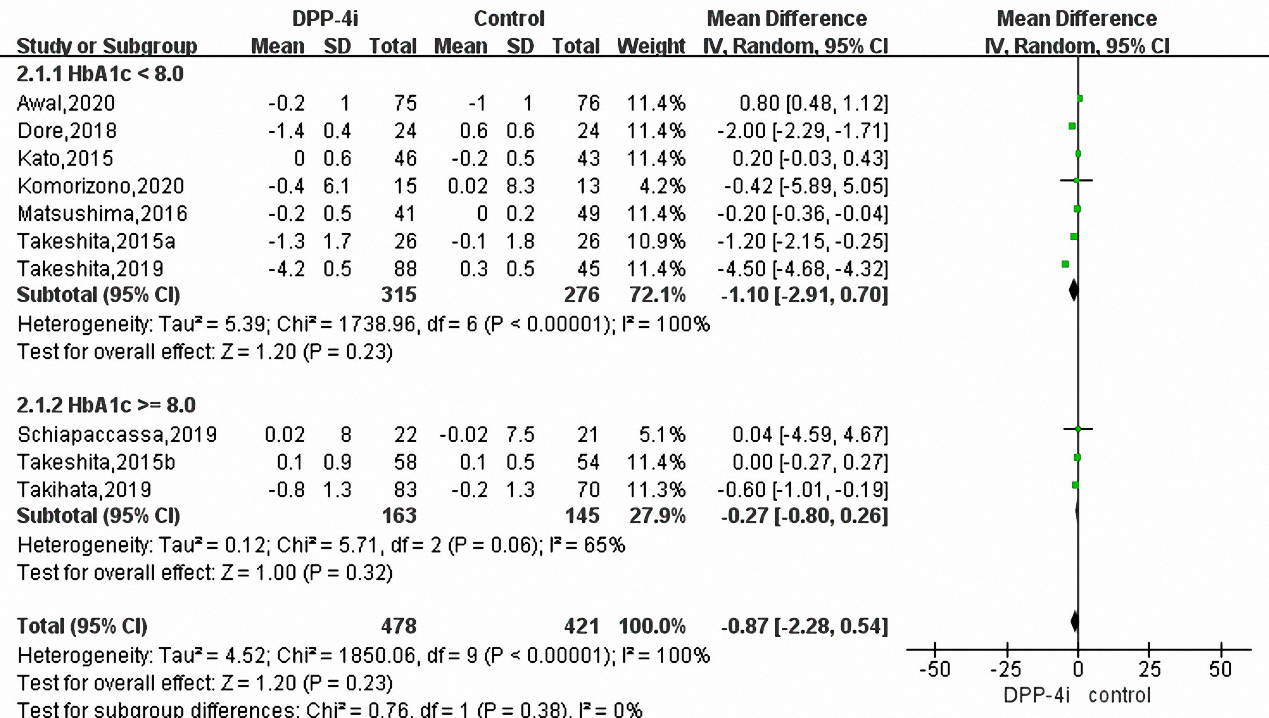


Supplementary figure 5 Subgroup analysis based on HbA1c.
